# Supplementary material for: Identifying immunologically-vulnerable regions of the HCV E2 glycoprotein and broadly neutralizing antibodies that target them
Source: Nat Commun. 2019 May 6;10:2073. doi: 10.1038/s41467-019-09819-1 (PMC6502829; doi:10.1038/s41467-019-09819-1)
Supplement: Supplementary file 1 — Supplementary Information [file 41467_2019_9819_MOESM1_ESM.pdf]

Supplementary Information

Identifying immunologically-vulnerable regions of the HCV E2 glycoprotein and broadly neutralizing antibodies that target them

Quadeer *et al.*

Table of contents

Supplementary Note 1

Supplementary Figures 1 – 12

Supplementary Tables 1 – 4

Supplementary References

Description of Additional Supplementary Files

Supplementary Notes

Supplementary Note 1. Comparison of predictions of the proposed model with those using a simpler conservation-only model

To investigate the importance of incorporating interactions between mutations at different residues, we compared the predictions of the proposed method with those obtained using a simpler model based only on amino acid conservation (or single mutant probabilities), which ignores such interactions. The most meaningful comparison is with respect to a conservation-based maximum entropy model parametrized only by the "fields" $h_i(a)$. These are given by

$$h_i(a) = \ln \frac{1 - f_i(a)}{f_i(a)} \ ,$$

where $f_i(a)$ is the frequency of observing mutant a at residue i. Our tests showed that, for the most direct biological validation of the model (i.e., comparison of model fitness predictions with in vitro infectivity measurements[1,2,11,3–10]), such a conservation-only model provided a much lower correlation with experimental fitness values ($\bar{r}$ = −0.64) (Supplementary Fig. 6; compare with the corresponding results for our model in Fig. 2a).

As we show below, further tests revealed that incorporating residue interactions into our model is not only important in making fitness predictions, but also provides interesting differences in the main results of the proposed work; namely, for the classification of antibodies based on relative escape times (Fig. 4a and Supplementary Fig. 4).

First, we computed the analogous result to Fig. 4a (relative escape times associated with the binding residues of HmAbs defined based on global alanine scanning[9]), using the conservation-only model. These results are shown in Supplementary Fig. 11, where they are contrasted against the predictions of our proposed model. A notable observation is that the predictions of the two models are quite distinct for domain D HmAb HC84-20. Specifically, while the conservation-only model predicted the minimum escape time of HC84-20 to be similar to the other domain D HmAbs (Supplementary Fig. 11, bottom panel), our model identified it as comparatively escape-resistant (Supplementary Fig. 11, top panel). This distinction in our model prediction suggests that the mutations at the non-conserved binding residues of HC84-20 still bear high escape time due to the additional constraints imposed by their interaction with other protein residues (that are taken into account exclusively in our model). Note that HmAb HC84-20 is different from the other domain D HmAbs (HC84-24 and HC84-26) as residue 442—a common binding residue of these HmAbs—is not a binding residue[9] of HC84-20 at RB $\leq 20\%$. Mutations at the residue 442 are known to be associated with escape from domain D HmAbs[12] and our model correctly associated it with relatively lower escape time (< 100 generations). This is evident from our model predictions at RB $\leq 40\%$ (Supplementary Fig. 4) where residue 442 is a binding residue of all domain D HmAbs (including HC84-20), and at RB $\leq 20\%$ (Supplementary Fig. 11, top panel) where it is a binding residue only of HmAbs HC84-24 and HC84-26. Thus, the absence of residue 442 from the binding residues of HmAb HC84-20 at RB $\leq 20\%$ suggests that this domain D HmAb may be relatively escape-resistant compared with other HmAbs, which is also in line with predictions of a recent report[13].

Second, we compared predictions of the two models for relative escape times associated with the binding residues of HmAbs defined based on selective alanine scanning (Supplementary Table 4). Contrary to the conservation-only model's prediction, our model predicted the minimum escape time of HmAbs AR3A-AR3C (Supplementary Fig. 12) to be comparatively higher than other HmAbs, suggesting the potential difficulty for the virus in escaping these antibodies. This is consistent with the reported potency of these antibodies in preventing as well as clearing chronic HCV infection in humanized mice[14].

Supplementary Figures

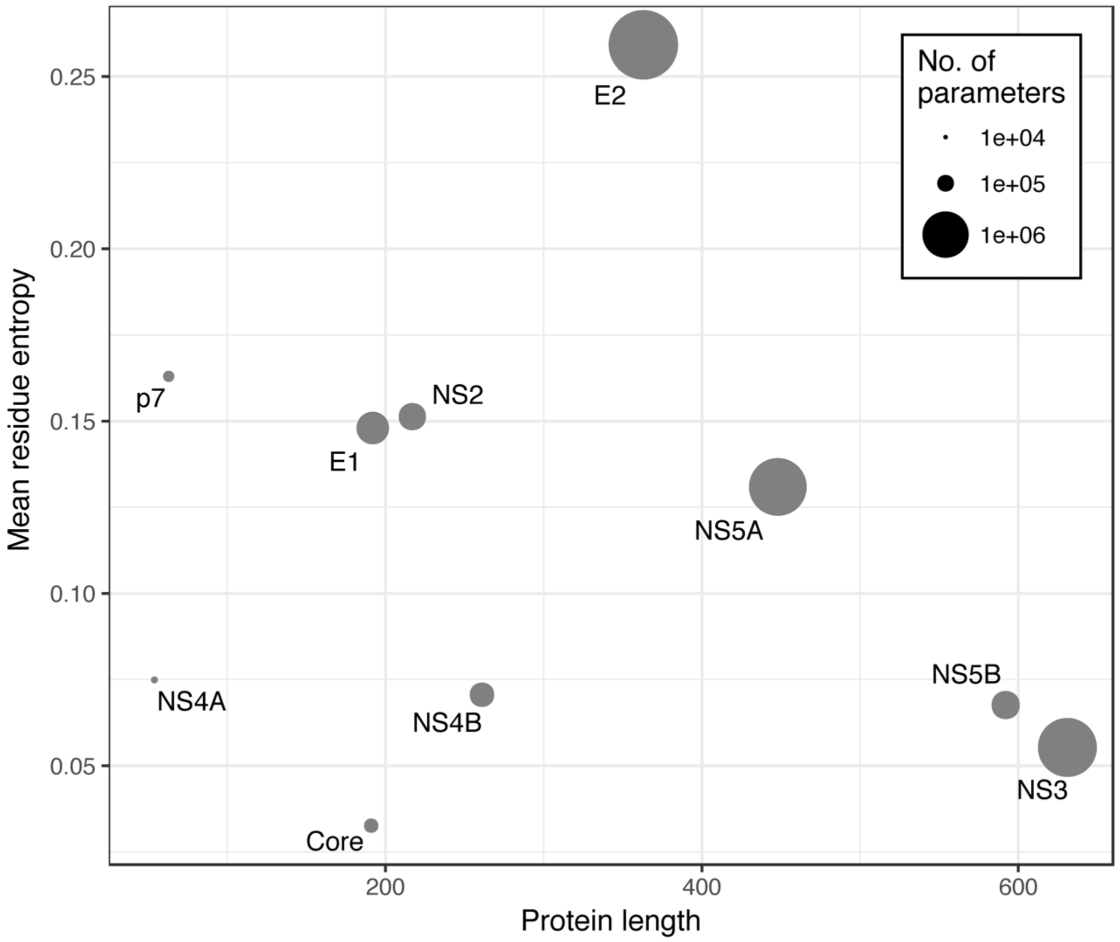

Supplementary Figure 1 | Comparison of the protein length, mean residue entropy, and number of parameters required to estimate fitness landscape for different HCV proteins. Of all HCV proteins, E2 has the highest mean residue entropy, as well as the highest number of parameters. Mean residue entropy H can be calculated by using the relation $H = \frac{1}{L}\sum_{i=1}^{L}\sum_{a=1}^{21} f_i(a)\ln f_i(a)$, where $f_i(a)$ is the frequency of observing an amino acid mutant a at residue i and L is the number of residues in the protein. The number of parameters was calculated by considering all the amino acid mutants observed at each residue.

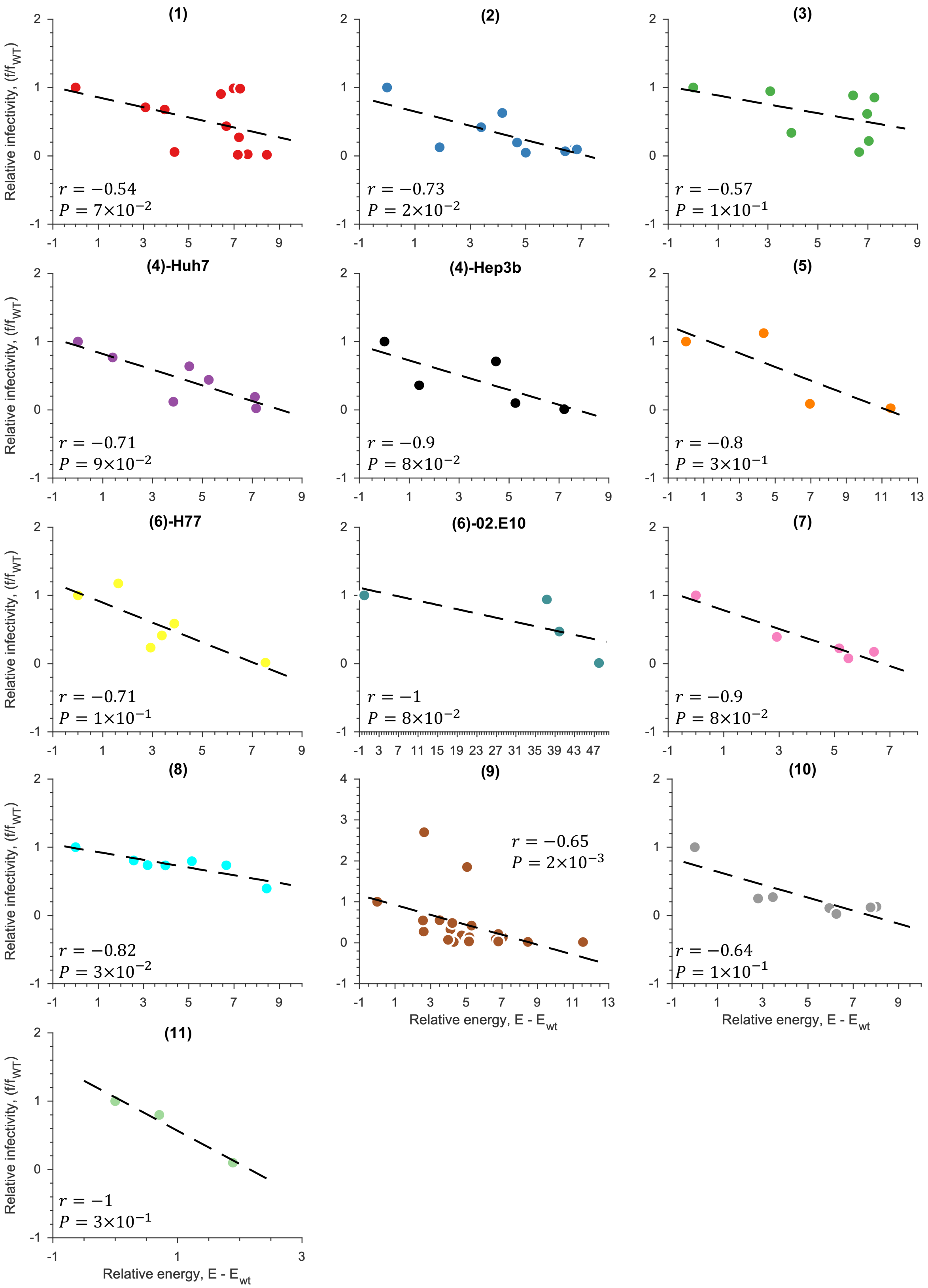

Supplementary Figure 2 | Fitness vs predicted energy for individual experiments compiled from the literature.

Both energy and infectivity are normalized with the reference strain used in the experiment. A relatively strong negative Spearman correlation is observed for all experiments. The reference associated with each set of experimental fitness values is shown above each subplot.

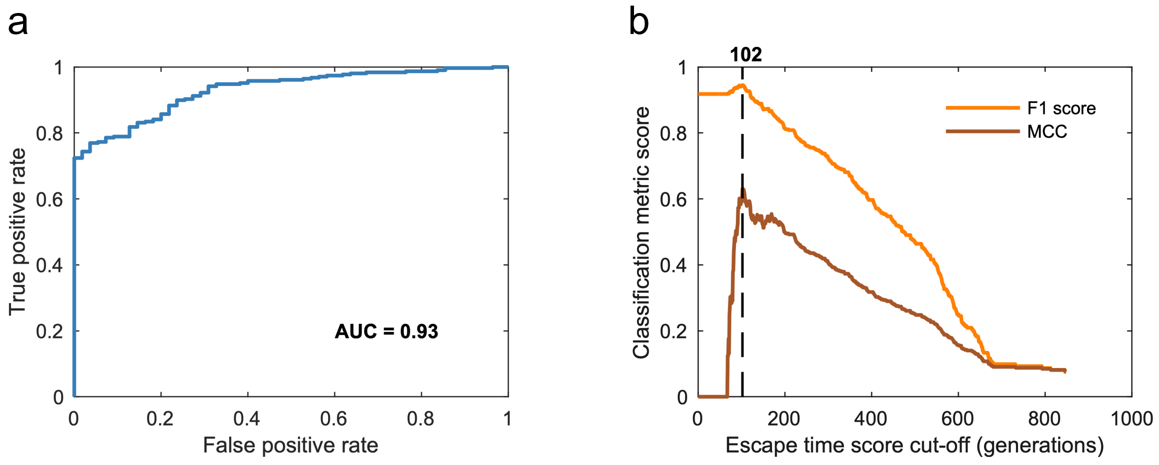

Supplementary Figure 3 | Classifier design based on the available information of experimentally/clinically observed escape mutations from E2-specific HmAbs.

a Receiver operating characteristic curve for identifying observed escape mutations (listed in Supplementary Table 2) using the inferred escape time scores. An area under the receiver operative characteristic curve (AUC) of 0.93 is avhieved. **b** Determination of the optimal escape time cut-off based on the F1 score and Matthews correlation coefficient (MCC). Note that in this binary classification, all residues at which escape mutation is not experimentally/clinically observed are considered difficult to escape.

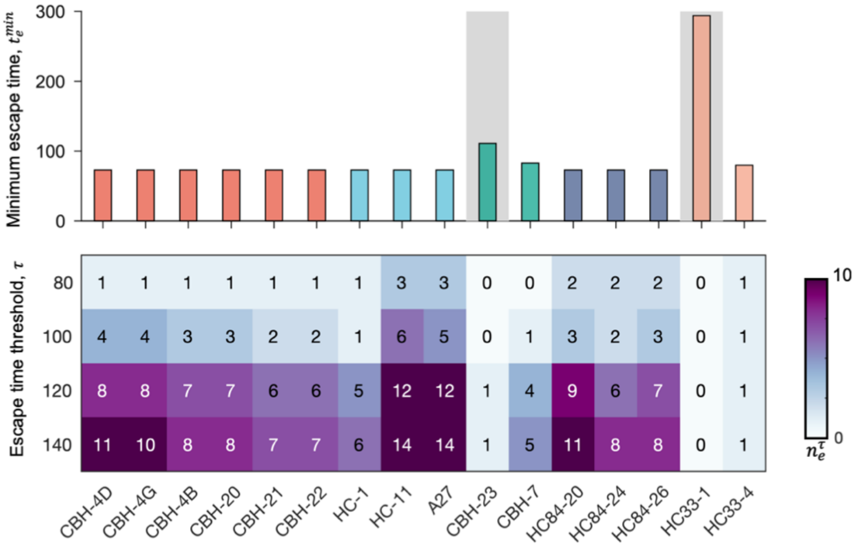

Supplementary Figure 4 | Analysis of relative escape times associated with mutating the binding residues (defined using RB $\leq 40\%$) of known HmAbs.

Minimum escape time t_e^{min} (top panel) and number of escape pathways n_e^{τ} (bottom panel) associated with mutating the binding residues of 16 HmAbs, determined using global alanine scanning[9]. The binding residues of the HmAbs are defined as the mutations that resulted in reduction of binding with respect to the wild-type to $\leq 40\%$. HmAbs are colored according to the antigenic domains using the scheme in Fig. 3b. The background of the HmAbs predicted to be relatively escape-resistant is shaded.

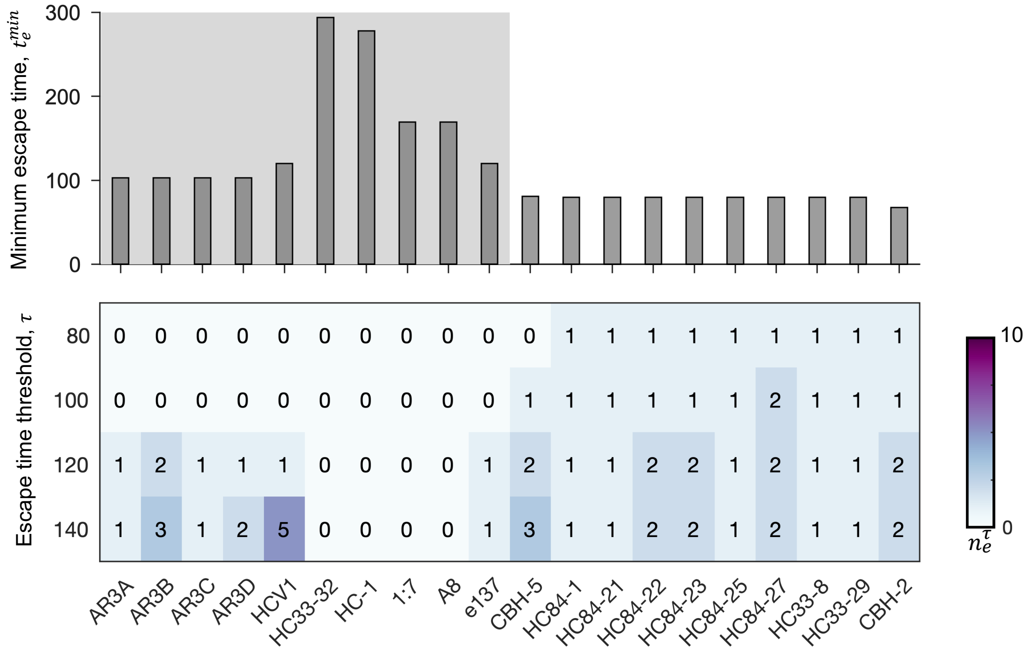

Supplementary Figure 5 | Analysis of relative escape times associated with mutating the binding residues (determined using selective alanine scanning mutagenesis) of known HmAbs.
Minimum escape time t_e^{min} (top panel) and number of escape pathways n_e^τ (bottom panel) associated with mutating the binding residues of HmAbs, where binding residues were determined using selective alanine scanning (Supplementary Table 4). All HmAbs predicted to be relatively escape-resistant are grouped together (in no specific order) on the left (shaded) and the remaining ones are grouped together on the right.

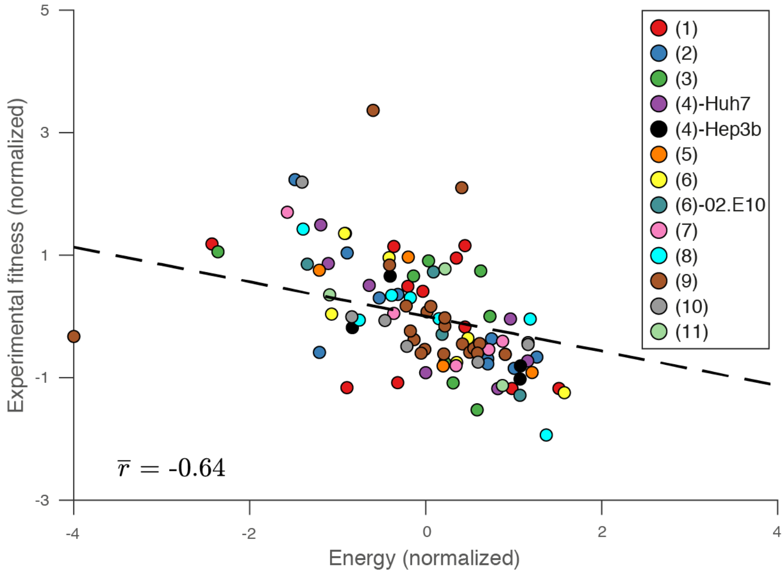

Supplementary Figure 6 | Experimental fitness vs energy for the conservation-only model.
Experimental fitness vs normalized energy (similar to Fig. 2a) for a maximum entropy model based only on single mutant probabilities (see Supplementary Note 1). The correlation $\bar{r}$ is the weighted Spearman correlation (see Methods).

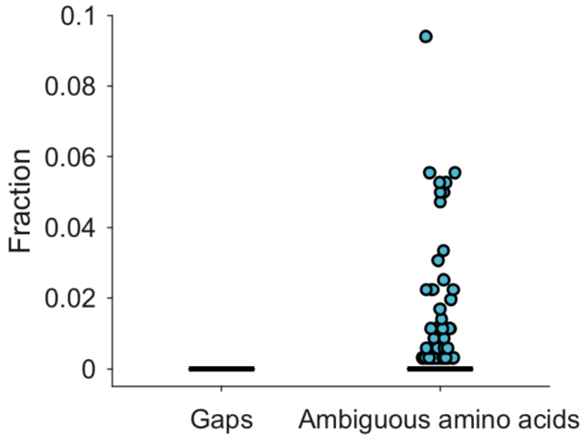

Supplementary Figure 7 | The fraction (per sequence) of gaps and ambiguous amino acids in the processed E2 sequence data.
In this box plot, the bold horizontal line indicates the median, the edges of the box represent the first and third quartiles, whiskers extend to span a 1.5 inter-quartile range from the edges (both box edges and whiskers are not visible due to the distribution being too skewed around the median), and the spheres represent the outliers (values beyond the range of whiskers).

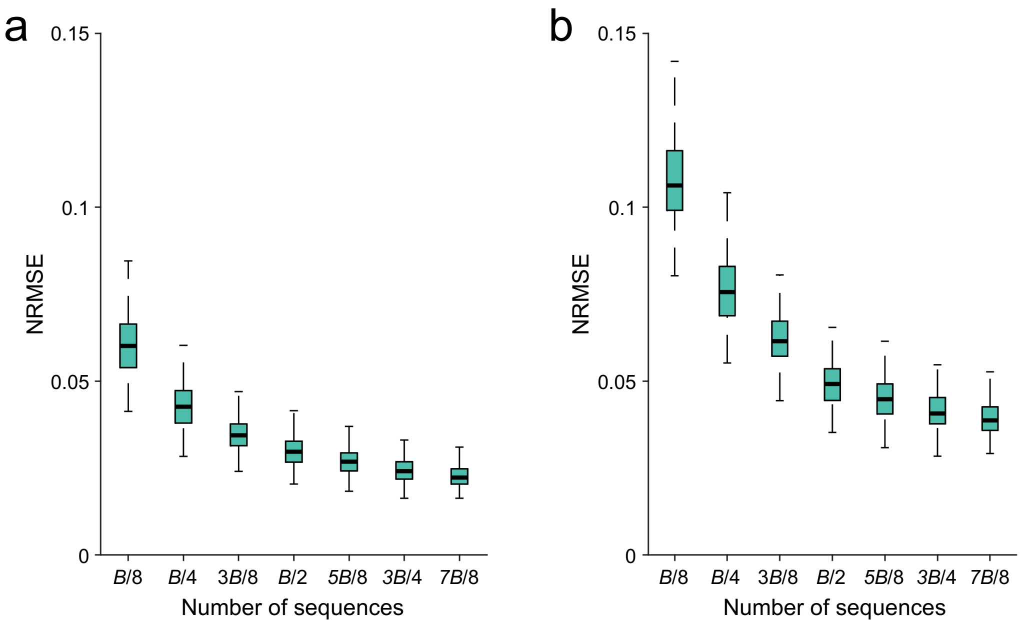

Supplementary Figure 8 | Robustness of the (a) single and (b) double mutation probabilities to the number of sequences in the MSA.
Each box plot shows the normalized root-mean-square-error (NRMSE) of the probabilities observed in the subsampled (sampling with replacement) MSA for 500 runs, where *B* is the total number of sequences (after data preprocessing). It can be observed that the median of the NRMSE of the probabilities calculated using the subsampled MSA converged to within 0.05, even when only half of the total sequences were used to construct the subsampled MSA. In each box plot, the bold horizontal line indicates the median, the edges of the box represent the first and third quartiles, and whiskers extend to span a 1.5 inter-quartile range from the edges.

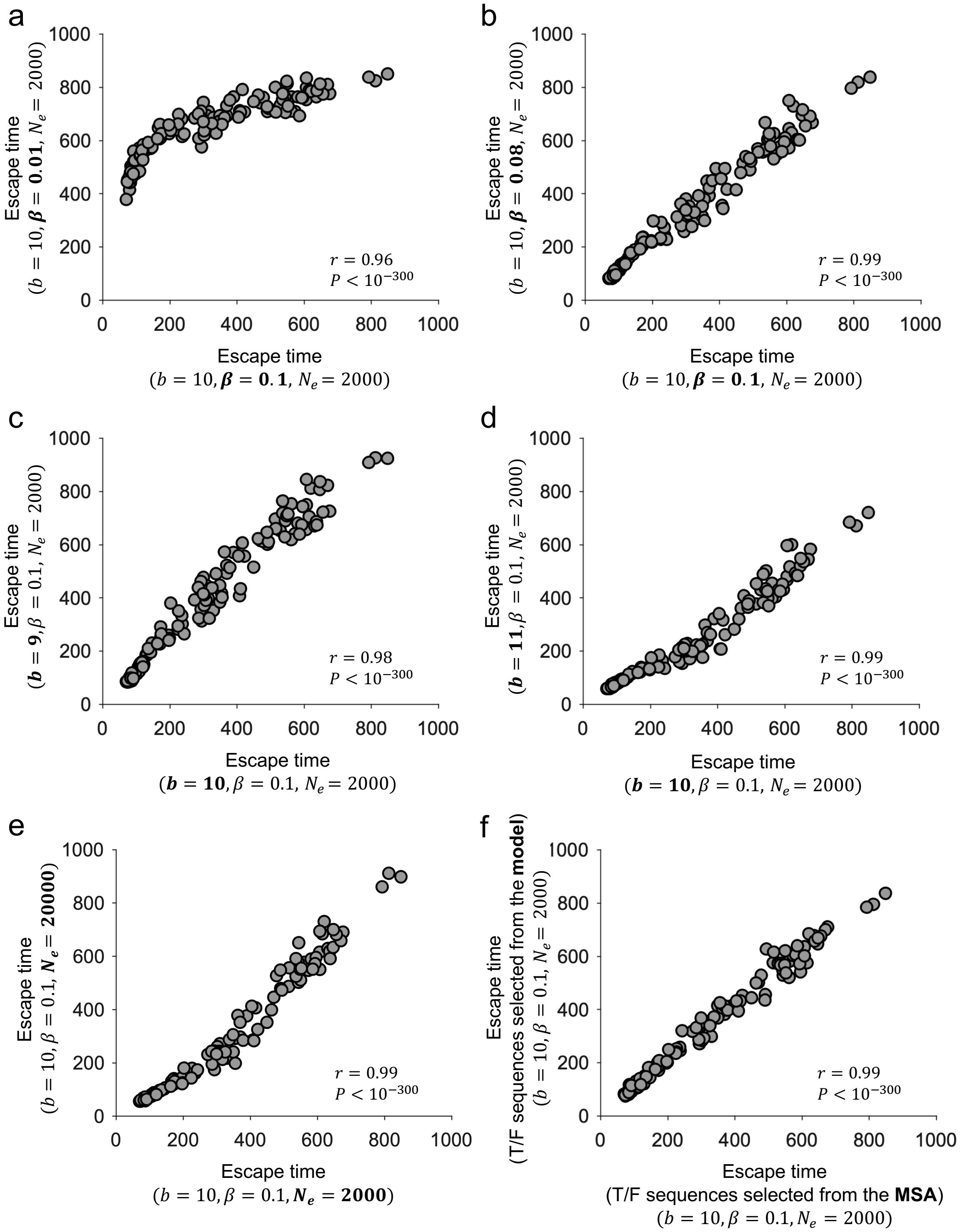

Supplementary Figure 9 | Robustness of escape time scores to the evolutionary model parameter values.
Comparison of predicted escape time scores for (**a–b**) different values of the parameter modeling the variation in viral fitness as function of predicted energy, β; for (**c–d**) different values of the parameter modeling the immune pressure, b; for (**e**) a different value of the effective population size, N_e; and for (**f**) different sets of T/F sequences employed. For (e), simulations with $N_e = 20{,}000$ were restricted to only 10 T/F sequences due to the very high computational complexity at this scale. For (f), escape time for each residue was computed by running simulations for 50 randomly sampled T/F sequences from the inferred model (equilibrium sampling via MCMC simulation). The main parameter differences in each subplot are shown in bold. The results are presented for the sets of residues involved in antigenic domains (Fig. 3b) or in forming the binding residues of HmAbs (Fig. 4a).

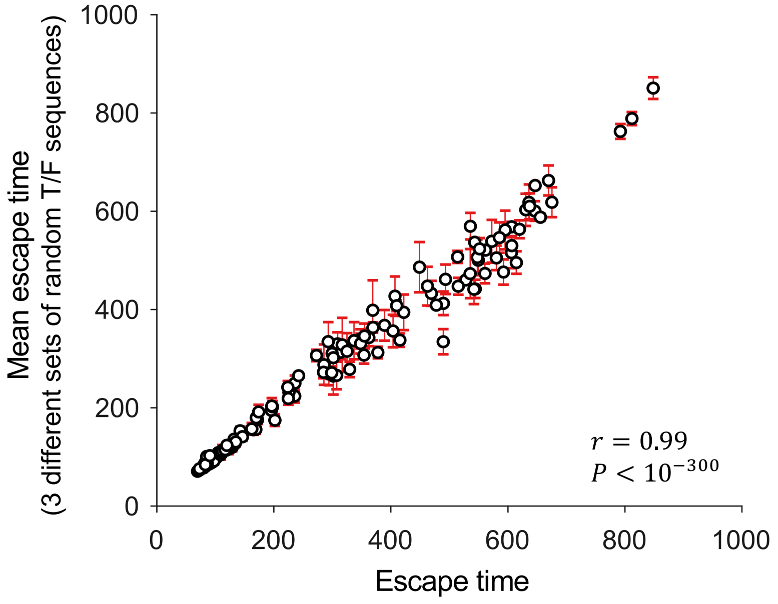

Supplementary Figure 10 | Robustness of escape time scores to different sets of T/F sequences used in the evolutionary simulations.
The y-axis represents the escape times averaged over three randomly-selected mutually distinct sets of 25 T/F sequences, with red error bars denoting one standard deviation. The result is presented for the sets of residues involved in antigenic domains (Fig. 3b) or in forming the binding residues of HmAbs (Fig. 4a).

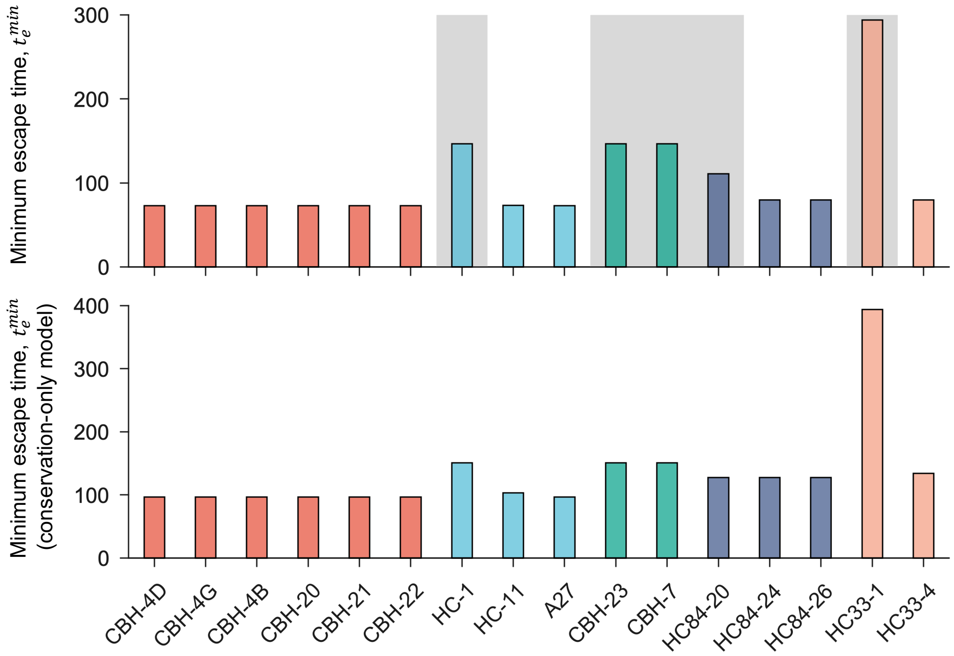

Supplementary Figure 11 | Comparison of predictions of the proposed model and the conservation-only model for the relative escape times associated with the binding residues of known HmAbs defined based on global alanine scanning.
Results using the proposed model (top panel) and the conservation-only model (bottom panel). The binding residues of the HmAbs are defined as the mutations that resulted in reduction of binding (RB) with respect to the wild-type to RB $\leq 20\%$. HmAbs are colored according to the antigenic domains using the scheme in Fig. 3b. The background of the HmAbs predicted by our proposed model to be relatively escape-resistant is shaded grey (top panel).

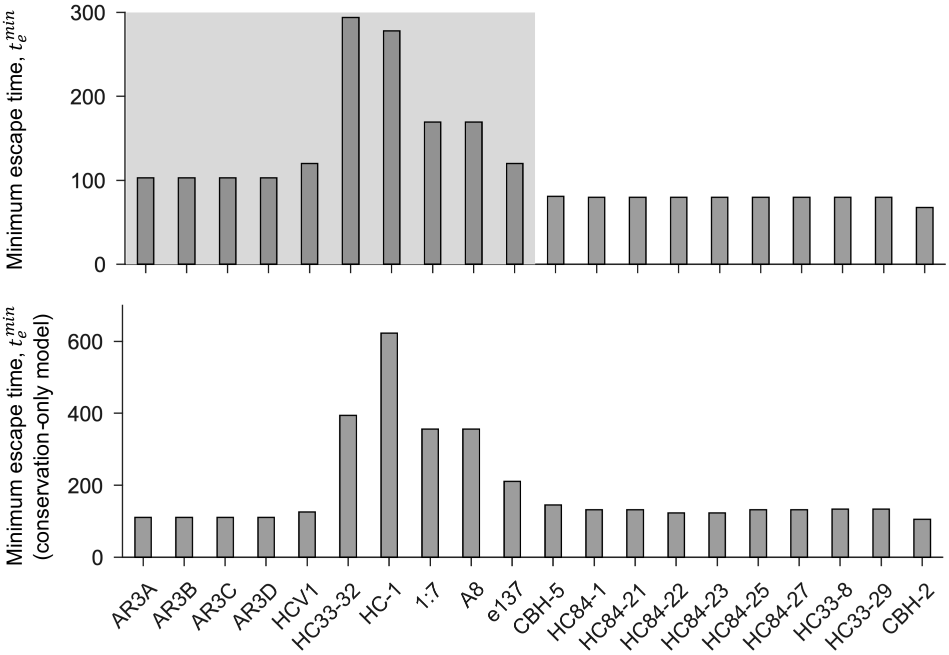

Supplementary Figure 12 | Comparison of predictions of the proposed model and the conservation-only model for the relative escape times associated with the binding residues of known HmAbs defined based on selective alanine scanning.
Results using the proposed model (top panel) and the conservation-only model (bottom panel). The binding residues were determined using selective alanine scanning mutagenesis (Supplementary Table 4). The background of the HmAbs predicted by our proposed model to be relatively escape-resistant is shaded grey (top panel).

Supplementary Tables

Supplementary Table 1. List of single mutations in H77 and H77 carrying N417S mutant background sequences that our model predicts to improve fitness.

Background sequence	Mutation	Decrease in energy (improvement in fitness)
H77	L399F	-0.3031
	T404A	-0.6243
	T404S	-0.4776
	E431D	-1.1979
	Q444H	-4.3557
	Q444R	-2.7261
	Q444V	-3.8673
	Q444Y	-3.0013
	L480P	-0.079
	A531E	-0.298
	L580R	-0.9659
H77 + N417S mutation	L399F	-0.3375
	V400A	-0.0818
	T404A	-0.3332
	T404S	-0.4024
	E431D	-1.4746
	Q444H	-4.552
	Q444R	-3.0376
	Q444V	-4.0852
	Q444Y	-3.1672
	A531E	-0.3078
	L580R	-1.2882

Supplementary Table 2. List of known escape mutations from E2-specific HmAbs.

Escape residues	HmAbs	Reference
431	CBH-2	[15]
431, 435, 444, 446, 466, 482, 501, 528, 531, 538, 580, 610, 636, 713	CBH-8C, CBH-2, CBH-5, HC-2, and HC-11	[6]
391,394, 401, 415, 417, 434, 444, 608	HCV1	[16]
416, 422, 424, 431, 433, 438, 442, 446, 453, 456, 461, 475, 482, 520, 524, 531, 533, 557, 558, 560	CBH-2, CBH-5, HC84-22, HC84-26, AR3A, AR3B, AR3C, AR3D	[12]
408	HC33-4	[17]
384, 386, 388, 390, 391, 393, 394, 395, 396, 397, 398, 399, 400, 401, 402, 403, 404, 405, 407, 410	HmAbs targeting HVR1	[18]

Supplementary Table 3. E2-restricted T lymphocyte epitopes available in the HIV Molecular Immunology Database[19].

Epitope	HLA Allele	Clinical outcome association
401–411	A2	N/A
459–507	B53	N/A
489–496	B51	Spontaneous clearance
530–539	B60	N/A
541–550	B57	Spontaneous clearance
569–578	B50	N/A
610–618	cw7	N/A
613–622	A2	N/A
621–628	A11	N/A
630–639	A3	Spontaneous clearance
632–640	A3	Spontaneous clearance
654–662	B60	N/A
686–694	A2	N/A
712–726	A24	N/A
723–734	A2	N/A

Supplementary Table 4. Binding residues of E2-specific HmAbs determined using selective alanine scanning mutagenesis.

Binding residues	HmAbs	Reference
424, 523, 525, 530, 535, 538, 540	AR3A	[20]
412, 416, 418, 423, 424, 523, 525, 530, 535, 540	AR3B	[20]
424, 488, 523, 525, 530, 535, 538, 540	AR3C	[20]
412, 424, 523, 530, 535	AR3D	[20]
412–423	HCV1	[16]
413, 418, 420	HC33-32	[21]
525, 530, 535	HC-1	[22]
485, 494, 497, 502, 503, 504, 505, 507, 508, 509, 523, 525, 530, 533, 535, 538, 540, 614, 617, 618, 619, 621, 624	CBH-5	[23,24]
523, 526, 527, 529, 530, 535	1:7	[25]
523, 526, 527, 529, 530, 535	A8	[25]
416, 420, 529, 530, 535	e137	[26]
441, 442	HC84-1	[7]
441–443	HC84-21	[7]
420, 428, 437, 441–443, 616	HC84-22	[7]
420, 428, 437, 441–443, 616	HC84-23	[7]
441, 442, 616	HC84-25	[7]
441–443, 446, 616	HC84-27	[7]
408, 413, 418, 420	HC33-8	[21]
408, 413, 418, 420	HC33-29	[21]
431, 437, 439, 530, 535, 523	CBH-2	[6,15]

Supplementary References

1. Goffard, A. *et al.* Role of N-linked glycans in the functions of hepatitis C virus envelope glycoproteins. *J. Virol.* **79,** 8400–8409 (2005).
2. Drummer, H. E., Boo, I., Maerz, A. L. & Poumbourios, P. A conserved Gly436-Trp-Leu-Ala-Gly-Leu-Phe-Tyr motif in hepatitis C virus glycoprotein E2 is a determinant of CD81 binding and viral entry. *J. Virol.* **80,** 7844–7853 (2006).
3. Falkowska, E., Kajumo, F., Garcia, E., Reinus, J. & Dragic, T. Hepatitis C virus envelope glycoprotein E2 glycans modulate entry, CD81 binding, and neutralization. *J. Virol.* **81,** 8072–8079 (2007).
4. Rothwangl, K. B., Manicassamy, B., Uprichard, S. L. & Rong, L. Dissecting the role of putative CD81 binding regions of E2 in mediating HCV entry: Putative CD81 binding region 1 is not involved in CD81 binding. *Virol. J.* **5,** 46 (2008).
5. Gal-Tanamy, M. *et al.* In vitro selection of a neutralization-resistant hepatitis C virus escape mutant. *Proc. Natl. Acad. Sci.* **105,** 19450–19455 (2008).
6. Keck, Z.-Y. *et al.* Mutations in hepatitis C virus E2 located outside the CD81 binding sites lead to escape from broadly neutralizing antibodies but compromise virus infectivity. *J. Virol.* **83,** 6149–6160 (2009).
7. Keck, Z. *et al.* Human monoclonal antibodies to a novel cluster of conformational epitopes on HCV E2 with resistance to neutralization escape in a genotype 2a isolate. *PLoS Pathog.* **8,** e1002653 (2012).
8. Guan, M. *et al.* Three different functional microdomains in the hepatitis C virus hypervariable region 1 (HVR1) mediate entry and immune evasion. *J. Biol. Chem.* **287,** 35631–35645 (2012).
9. Pierce, B. G. *et al.* Global mapping of antibody recognition of the hepatitis C virus E2 glycoprotein: Implications for vaccine design. *Proc. Natl. Acad. Sci.* **113,** E6946–E6954 (2016).
10. Gopal, R. *et al.* Probing the antigenicity of hepatitis C virus envelope glycoprotein complex by high-throughput mutagenesis. *PLoS Pathog.* **13,** 1–27 (2017).
11. El-Diwany, R. *et al.* Extra-epitopic hepatitis C virus polymorphisms confer resistance to broadly neutralizing antibodies by modulating binding to scavenger receptor B1. *PLoS Pathog.* **13,** e1006235 (2017).
12. Bailey, J. R. *et al.* Naturally selected hepatitis C virus polymorphisms confer broad neutralizing antibody resistance. *J. Clin. Invest.* **125,** 437–447 (2015).
13. Cowton, V. M., Singer, J. B., Gifford, R. J. & Patel, A. H. Predicting the effectiveness of hepatitis C virus neutralizing antibodies by bioinformatic analysis of conserved epitope residues using public sequence data. *Front. Immunol.* **9,** 1470 (2018).
14. de Jong, Y. P. *et al.* Broadly neutralizing antibodies abrogate established hepatitis C virus infection. *Sci. Transl. Med.* **6,** 254ra129-254ra129 (2014).
15. Keck, Z.-Y. *et al.* A point mutation leading to hepatitis C virus escape from neutralization by a monoclonal antibody to a conserved conformational epitope. *J. Virol.* **82,** 6067–6072 (2008).
16. Morin, T. J. *et al.* Human monoclonal antibody HCV1 effectively prevents and treats HCV infection in chimpanzees. *PLoS Pathog.* **8,** e1002895 (2012).
17. Keck, Z. *et al.* Antibody response to hypervariable region 1 interferes with broadly neutralizing antibodies to hepatitis C virus. *J. Virol.* **90,** 3112–3122 (2016).
18. Kato, N., Sekiya, H. & Ootsuyama, Y. Humoral immune response to hypervariable region 1 of the putative envelope glycoprotein (gp70) of hepatitis C virus. *J. Virol.* **67,** 3923–3930 (1993).
19. Yusim, K. et al. (eds.) *HIV Molecular Immunology* (Los Alamos National Laboratory, Theoretical Biology and Biophysics, Los Alamos, New Mexico, 2016). LA-UR 17-24847. https://www.hiv.lanl.gov/content/immunology/index.html
20. Law, M. *et al.* Broadly neutralizing antibodies protect against hepatitis C virus quasispecies challenge. *Nat. Med.* **14,** 25–27 (2008).
21. Keck, Z. *et al.* Cooperativity in virus neutralization by human monoclonal antibodies to two adjacent regions located at the amino terminus of hepatitis C virus E2 glycoprotein. *J. Virol.* **87,** 37–51 (2013).
22. Keck, Z.-Y. *et al.* Mapping a region of hepatitis C virus E2 that is responsible for escape from neutralizing antibodies and a core CD81-binding region that does not tolerate neutralization escape mutations. *J. Virol.* **85,** 10451–10463 (2011).
23. Owsianka, A. M. *et al.* Broadly neutralizing human monoclonal antibodies to the hepatitis C virus E2 glycoprotein. *J. Gen. Virol.* **89,** 653–659 (2008).
24. Iacob, R. E., Keck, Z., Olson, O., Foung, S. K. H. & Tomer, K. B. Structural elucidation of critical residues involved in binding of human monoclonal antibodies to hepatitis C virus E2 envelope glycoprotein. *Biochim. Biophys. Acta - Proteins Proteomics* **1784,** 530–542 (2008).
25. Johansson, D. X. *et al.* Human combinatorial libraries yield rare antibodies that broadly neutralize hepatitis C virus. *Proc. Natl. Acad. Sci.* **104,** 16269–16274 (2007).
26. Perotti, M. *et al.* Identification of a broadly cross-reacting and neutralizing human monoclonal antibody directed against the hepatitis C virus E2 protein. *J. Virol.* **82,** 1047–1052 (2008).

Description of Additional Supplementary Files

File Name: Supplementary Data 1
Description: The mean escape time predicted for each residue in E2.

File Name: Supplementary Data 2
Description: Accession numbers of E2 sequences used for inferring the model.

File Name: Supplementary Data 3
Description: The experimental fitness (infectivity) measurements for E2 compiled from the literature.
