# Supplementary material for: Identifying immunologically-vulnerable regions of the HCV E2 glycoprotein and broadly neutralizing antibodies that target them
Source: Nat Commun. 2019 May 6;10:2073. doi: 10.1038/s41467-019-09819-1 (PMC6502829; doi:10.1038/s41467-019-09819-1)
Supplement: Supplementary file 3 — Reporting Summary [file 41467_2019_9819_MOESM3_ESM.pdf]

nature research

Corresponding author(s): Matthew R. McKay

Last updated by author(s): Mar 26, 2019

Reporting Summary

Nature Research wishes to improve the reproducibility of the work that we publish. This form provides structure for consistency and transparency in reporting. For further information on Nature Research policies, see Authors & Referees and the Editorial Policy Checklist.

Statistics

For all statistical analyses, confirm that the following items are present in the figure legend, table legend, main text, or Methods section.

n/a	Confirmed	
☐	☒	The exact sample size (n) for each experimental group/condition, given as a discrete number and unit of measurement
☐	☒	A statement on whether measurements were taken from distinct samples or whether the same sample was measured repeatedly
☐	☒	The statistical test(s) used AND whether they are one- or two-sided<br>*Only common tests should be described solely by name; describe more complex techniques in the Methods section.*
☒	☐	A description of all covariates tested
☐	☒	A description of any assumptions or corrections, such as tests of normality and adjustment for multiple comparisons
☐	☒	A full description of the statistical parameters including central tendency (e.g. means) or other basic estimates (e.g. regression coefficient) AND variation (e.g. standard deviation) or associated estimates of uncertainty (e.g. confidence intervals)
☐	☒	For null hypothesis testing, the test statistic (e.g. F, t, r) with confidence intervals, effect sizes, degrees of freedom and P value noted<br>*Give P values as exact values whenever suitable.*
☐	☒	For Bayesian analysis, information on the choice of priors and Markov chain Monte Carlo settings
☐	☒	For hierarchical and complex designs, identification of the appropriate level for tests and full reporting of outcomes
☐	☒	Estimates of effect sizes (e.g. Cohen's d, Pearson's r), indicating how they were calculated

Our web collection on statistics for biologists contains articles on many of the points above.

Software and code

Policy information about availability of computer code

Data collection	E2 1a sequences, used for inferring the model, were downloaded from the publicly available LANL HCV sequence database, https://hcv.lanl.gov. Specifically, genotype "1", subtype "a", and genomic region "E2" was searched at https://hcv.lanl.gov/components/sequence/HCV/search/searchi.html. The experimental fitness (infectivity) measurements for E2 were compiled from multiple literature reports.
Data analysis	The software implementation of the minimum-probability-flow-based method, used for inferring the fitness landscape parameters, is available at https://github.com/raymondlouie/MPF-BML. Data and scripts for reproducing the results are available at https://github.com/ahmedaq/HCV-E2.

For manuscripts utilizing custom algorithms or software that are central to the research but not yet described in published literature, software must be made available to editors/reviewers. We strongly encourage code deposition in a community repository (e.g. GitHub). See the Nature Research guidelines for submitting code & software for further information.

Data

Policy information about availability of data

All manuscripts must include a data availability statement. This statement should provide the following information, where applicable:

- Accession codes, unique identifiers, or web links for publicly available datasets
- A list of figures that have associated raw data
- A description of any restrictions on data availability

The mean escape time predicted for each residue in E2 is summarized in Supplementary Data 1. Accession numbers of E2 1a sequences used for inferring the model are listed in Supplementary Data 2. The experimental fitness (infectivity) measurements for E2 compiled from literature are included in Supplementary Data 3.

Field-specific reporting

Please select the one below that is the best fit for your research. If you are not sure, read the appropriate sections before making your selection.

☐ Life sciences ☐ Behavioural & social sciences ☒ Ecological, evolutionary & environmental sciences

For a reference copy of the document with all sections, see nature.com/documents/nr-reporting-summary-flat.pdf

Ecological, evolutionary & environmental sciences study design

All studies must disclose on these points even when the disclosure is negative.

Study description	We employed a maximum-entropy-based method to infer an in silico model for the fitness landscape of the HCV E2 protein using available sequences for genotype 1a. We then integrated the fitness landscape into a stochastic population genetics model of in-host viral evolution, which we employed to quantify the average time to escape from antibody responses targeting any specific residue in E2.
Research sample	We downloaded the amino acid multiple sequence alignment (MSA) of HCV genotype 1a E2 sequences from the Los Alamos National Laboratory (LANL) HCV sequence database (https://hcv.lanl.gov; accessed Sep. 25, 2017).
Sampling strategy	N/A
Data collection	N/A
Timing and spatial scale	The downloaded data includes sequences from multiple patients collected from 1977 - 2014.
Data exclusions	To control sequence quality, we excluded (i) the problematic sequences (default option) which include sequences that are either very short, comprise large deletions, or are artificially synthesized in laboratories; and (ii) the sequences having more than 2% gaps in the MSA.
Reproducibility	Data and scripts for reproducing the results are available at https://github.com/ahmedaq/HCV-E2.
Randomization	N/A
Blinding	N/A

Did the study involve field work? ☐ Yes ☒ No

Reporting for specific materials, systems and methods

We require information from authors about some types of materials, experimental systems and methods used in many studies. Here, indicate whether each material, system or method listed is relevant to your study. If you are not sure if a list item applies to your research, read the appropriate section before selecting a response.

Materials & experimental systems

n/a	Involved in the study	
☒	☐	Antibodies
☒	☐	Eukaryotic cell lines
☒	☐	Palaeontology
☒	☐	Animals and other organisms
☒	☐	Human research participants
☒	☐	Clinical data

Methods

n/a	Involved in the study	
☒	☐	ChIP-seq
☒	☐	Flow cytometry
☒	☐	MRI-based neuroimaging
