# Supplementary material for: Identifying immunologically-vulnerable regions of the HCV E2 glycoprotein and broadly neutralizing antibodies that target them
Source: Nat Commun. 2019 May 6;10:2073. doi: 10.1038/s41467-019-09819-1 (PMC6502829; doi:10.1038/s41467-019-09819-1)
Supplement: Supplementary file 4 — Description of Additional Supplementary Files [file 41467_2019_9819_MOESM4_ESM.pdf]

Description of Additional Supplementary Files

File Name: Supplementary Data 1
Description: The mean escape time predicted for each residue in E2.

File Name: Supplementary Data 2
Description: Accession numbers of E2 sequences used for inferring the model.

File Name: Supplementary Data 3
Description: The experimental fitness (infectivity) measurements for E2 compiled from the literature.
